# Advancing environmental exposure assessment science to benefit society

**DOI:** 10.1038/s41467-019-09155-4

**Published:** 2019-03-15

**Authors:** Andrew Caplin, Masoud Ghandehari, Chris Lim, Paul Glimcher, George Thurston

**Affiliations:** 1 School of Arts and Sciences, Department of Economics, New York University, New York, NY USA; 2 Tandon School of Engineering, Department of Urban Engineering, New York University, New York, NY USA; 3 NYU School of Medicine, Department of Environmental Medicine, New York University, New York, NY USA

## Abstract

Awareness of the human health impacts of exposure to air pollution is growing rapidly. For example, it has become evident that the adverse health effects of air pollution are more pronounced in disadvantaged populations. Policymakers in many jurisdictions have responded to this evidence by enacting initiatives that lead to lower concentrations of air pollutants, such as urban traffic restrictions. In this review, we focus on the interplay between advances in environmental exposure assessment and developments in policy. We highlight recent progress in the granular measurement of air pollutants and individual-level exposures, and how this has enabled focused local policy actions. Finally, we detail an illustrative study designed to link individual-level health-relevant exposures with economic, behavioral, biological, familial, and environmental variables.

## Abstract (web summary)

How can scientists and policymakers work together to reduce the health impacts of air pollution? In this review paper, the authors discuss the interplay between advances in environmental exposure assessment and policy advances to tackle pollution in a focused way.

## Introduction

Awareness of the human health impacts of exposure to air pollution is growing rapidly^1–5^, and is increasingly being translated into public policy^6,7^. A particular spur to action is that the effects of air pollution are often most clearly evidenced among the most disadvantaged populations^8–11^. For example, children in households with low-socioeconomic status (SES) have worse health at birth than those with high SES^12^. This gradient between SES and health steepens during life, and various health conditions such as respiratory disease can attribute to this relationship^13^. As a respiratory disease, childhood asthma can be aggravated and induced by air pollution exposure^14,15^. Those who are less well-off often live in areas where the air contains higher levels of hazardous particulate matter (PM)^16,17^, thus having a compound effect on health conditions later in life. While all are of us are affected by air pollution exposure, the combination of compromised baseline health and lower air quality can inflict even more profound long-term health effects^18^.

Given this sobering reality, policymakers in many countries and communities have begun responding appropriately by controlling or banning dangerous air pollutants, requiring cleaner heating fuels, restricting urban traffic, etc. The political will for cleaner air presents us with a tremendous opportunity. The more that factors determining pollutant exposures are detailed, the better individuals and policymakers can mitigate elevated exposure and adverse health effects. In this paper, we focus precisely on the interplay between advances in environmental exposure assessment on the one hand, and policy advances on the other.

In the past, pollution measurement has been relatively crude in terms of compositional, spatial, and temporal resolution. There have been significant improvements in the ability to conduct granular measurement of air pollutants, and this has enabled more awareness and more focused local policy actions, as addressed below. We outline more recent progress and the most current practices in the ability to measure pollution exposure at the individual level, rather than in geographical terms. The level of practical use of these personal monitoring methods has not yet been developed to the point of directly impacting policy (e.g., by their application for ambient air regulatory purposes), but the potential is high. We detail an illustrative study designed to turn that potential into practice by measuring individuals’ exposures over a prolonged period of time, along with measures of factors that influence health outcomes, including economic, behavioral, biological, familial, and environmental contexts. Though other parallel projects may well be in planning elsewhere, we describe this initiative, which is currently being applied in New York City, as a pilot study for the method applied in a very large metropolitan area. We expect that such individual-level studies will deepen the understanding of the health outcomes of pollution exposure, enable personal preventive actions, motivate collective adjustment, thereby enabling more effective policies. There are challenges in enabling policies that limit personal exposures, but the likely improvements in health and welfare are potentially massive.

## Pollution and poverty

Common outdoor “community”-based air pollutants that have been identified as associated with increasing risks of cardiovascular and respiratory diseases, lung cancer, and early death, include ozone (O_3_); fine PM_2.5_ and elemental carbon (EC) soot; nitrogen dioxide (NO_2_); and sulfur dioxide (SO_2_)^19^. Recently, increased attention has also been directed toward ultrafine particles, defined as particles with ≤0.1 µm in aerodynamic diameter, as they also have been associated with adverse health effects, largely independent of PM_2.5_ mass^20,21^. Elevated exposures to PM_2.5_ are especially widespread^22^, with among the most dangerous of these apparently being from fossil fuel combustion, especially coal burning^23–25^, likely because of these particles’ small size and especially toxic composition. These exposures have been estimated to shorten adult life expectancy by 1–2 years^26^, while also damaging long-term cardiac and pulmonary health^27,28^. Recent evidence also implicates exposure to a range of adverse health outcomes, including neurological diseases, cardio-metabolic diseases, and renal disease^29–31^.

Understanding of the widespread impact of air pollution on human health, and the associated economic impact, has improved in recent years^32–34^. For example, estimates by Public Health England have indicated that the exposure to PM and nitrogen dioxide air pollution in London resulted in 41,404 life years lost in 2010, with an estimated associated annual financial impact of £3,653 million^35^. Worldwide, WHO has estimated that each year, more than 7 million premature deaths can be attributed to the combined effects of indoor and outdoor air pollution^36^. Overall, 80% of the premature deaths attributed to outdoor air pollution are caused by ischemic heart disease and strokes, 14% are due to chronic obstructive pulmonary disease or acute lower respiratory infections, and 6% are due to lung cancer, primarily from exposure to fine PM_2.5_. A recent Global Burden of Disease (GBD) study reported that 4.2 million annual deaths were attributable to ambient PM_2·5_, making it the fifth-ranking mortality risk factor in 2015 globally, and that, if trends of increasing levels of air pollution continue in low- and middle-income countries, the increasing health burdens will be significant, if a substantial reduction of pollution is not achieved.^37^. Compounding this concern, a recent reanalysis of available air pollution cohort studies has revealed that past GBD estimates of the mortality effects, which relied on the integrated exposure-response, have been underestimating the effects of outdoor air pollution on human mortality^38^.

Of the health problems known to be worsened by air pollution, asthma is among the most widespread, especially among the children. A study looking at asthma prevalence and health care in the U.S. found that, in 2008, persons with asthma missed 10.5 million school days and 14.2 million work days due to their asthma and that, in 2007, there were 1.75 million asthma-related emergency department visits and 456,000 asthma hospitalizations^39^. A majority of childhood asthma is estimated to be induced by environmental exposures^40,41^. In 1 year alone, the cost of childhood exposure to environmental contaminants resulting in asthma was estimated to be close to 2 billion USD^34^. Several recent reviews have found evidence for a link between air pollution and incidence of new cases of child and adult asthma^15,42,43^, however, the evidence has still been considered by some as yet insufficient proof of a causal link.

Poverty is known to be a critical factor in susceptibility to pollution-related asthma; a 1999 study in Los Angeles found that the air pollution-related asthma admissions were higher for families of lower income^44^, independent of health insurance coverage. Similarly, an analysis of records of daily hospital admissions and air pollution in New York City (NYC), established that an apparent difference between the races in the risk of hospital admissions was explained by consideration of lower SES, where the poor and the working poor were found to have higher risks per amount of pollution exposure than wealthier individuals, irrespective of race^45^. Also, a recent US study of some 60 million older adults found that the mortality effects from long-term air pollution exposure in the elderly Medicare population was greater amongst those eligible for Medicaid (i.e., those with lower income) than others in that population^46^, again indicating that SES is a primary risk factor that increases susceptibility to adverse health outcomes of air pollution.

Other studies expand on the link between SES status and health effects of pollution. As reported for Sweden^47^, fluctuations in pollution caused by atmospheric inversions were used as indicators of pollution effects on health, while considering the role of SES. Inversions are transient episodes when the usual gradient of near surface air temperature (cooling with increasing elevation) reverses, resulting in an atmospheric trapping of pollutants. The authors used information on pollution, weather, and precipitation generated by NASA and the Swedish Environmental Research Institute, as well as pollution measured at fixed sites (typically located in town centers in Sweden). They acquired health data from *Statistics Sweden*, as well as inpatient and outpatient data covering all those of age 18 or under. The authors found that the worsening of pollution associated with atmospheric inversions increased health care visits for respiratory illnesses, and that the impact on children from high-income households was significantly lower than that on children in low-income households, indicating that baseline health played a key role. While the impact was virtually identical across income groups for children in poor health, among children in good health the impact of pollution was much larger for low and medium income than for high-income families.

## Pollution policy at the global and national level

Air pollution exposure is a global health concern: some 90% of the world’s population is breathing outdoor air that falls below the World Health Organization (WHO) air quality guidelines^36^. In the US alone, more than 125 million people are estimated to live in communities with unhealthy air^48^. In the developing world, while accelerated economic development and industrialization has led to a reduction of poverty, it has also resulted in greater economic disparities and extreme poor air qualities, especially in megacities^49^. There are many health risks and economic impacts associated with such pollution exposures, including a diminished lifelong economic productivity that results from permanent reduction in physical health and cognitive capability, compounded by increased costs of health care and early morbidity^34^.

Knowledge of the damage that pollutants impose on health has been instrumental in policies and global accords; the Paris Agreement being one example. Recent progress in the scientific understanding of pollution, and the air quality implications of anthropogenic climate change mitigation policies, highlight the progress for cleaner air can immediately benefit society at large^50^.

## Improving air pollution policy at the local level

**Fig. 1:**
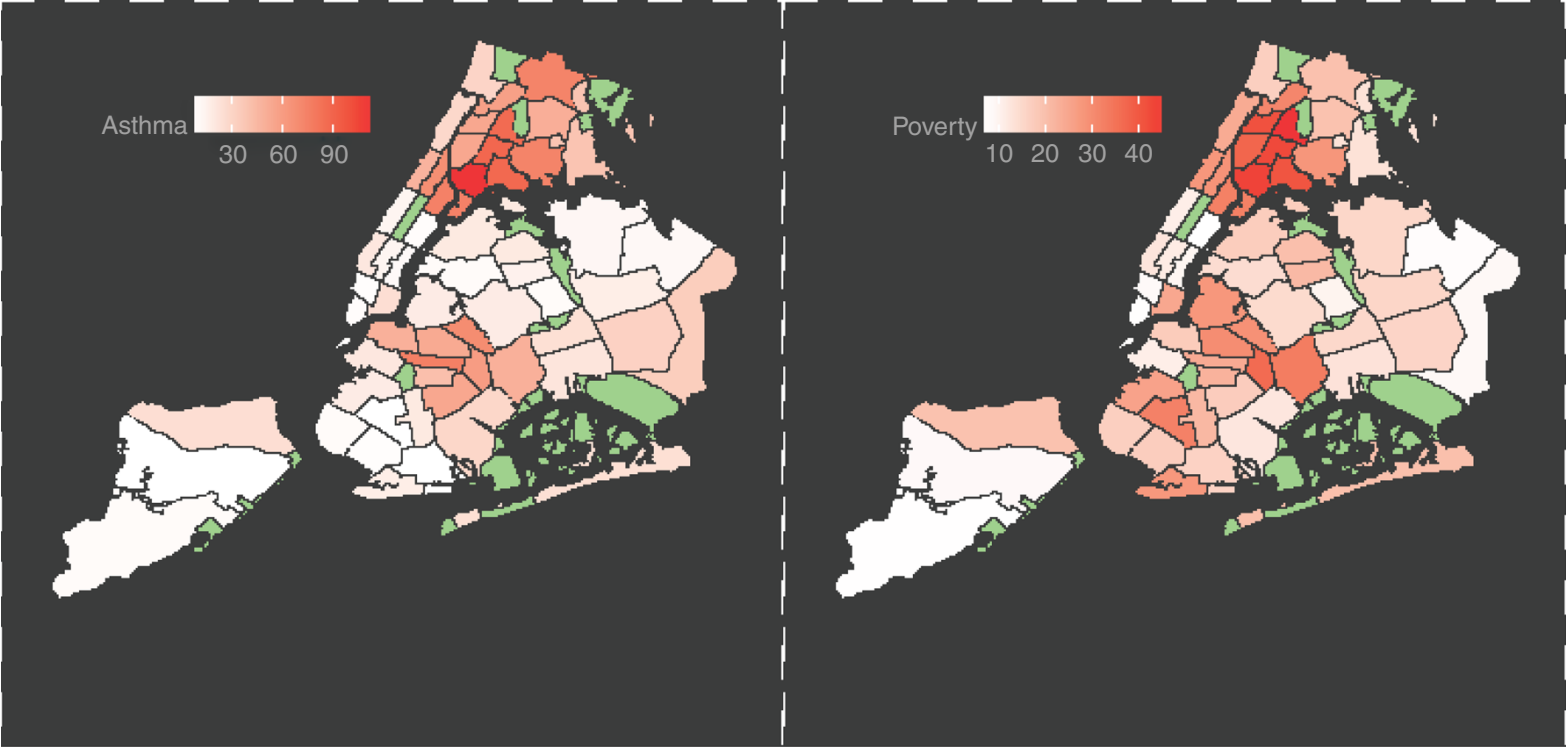
NYC 2014 Community District Data for: (left) Children’s Asthma Admissions per 10,000 persons; and, (right) percent poverty in the same period. Green shaded areas represent parks, airports, etc. Figure rendered using data from NY State DOH^6^

While there have been striking air quality policy successes in the past, such as the US Clean Air Act, there are limitations as to what can be achieved when relying only on traditional central-site air quality measurements that often deliver only infrequent measures of just a few select pollutants. It is now known that air pollution, and its composition, often varies dramatically over short distances and periods of time^51,52^. Likewise, awareness has grown regarding the links between pollution and poverty. The geographic link between asthma and poverty in NYC is one example, as indicated in Fig. 1, showing: (a) the spatial distribution of asthma admissions rates by children aged 5–14 (per 10,000 population) across the 59 Community Districts in NYC during 2010–2014; and (b) the percent of poverty in the population in each of the same Districts in the same time period. The spatial correlation between poor health and poverty is apparent; a statistical analysis of these data also indicates a significant association between health and wealth in this case (*r* = 0.35, *p* = 0.01).

In Fig. 1, note that the asthma admissions rates vary dramatically across New York City. This suggests that it is important to understand local patterns of pollution exposures. Unfortunately, it has been common to measure pollutant exposure levels to represent large and highly heterogeneous areas (e.g., city or county-wide). The fixed monitoring stations that are used in the U.S. mainly for regulatory purposes are often sparsely located, and elevated on buildings. New York City, for example, has 13 high-performance regulatory air monitoring stations. While this level of instrumentation has been of great value, and has resulted in positive action, differences in air quality across individual neighborhoods, and at ground-level, are not captured by such coarsely distributed and elevated air monitors.

The chemical constituents and sources of pollution are also often studied with low specificity. The label “fine particulate matter mass” is highly nonspecific, even though the mass is comprised of many different particles with differing compositions and sources that can have differing health implications. For example, studies have been carried out on differentiation of outdoor PM_2.5_ (fine mass) exposures across the US by composition and source category^53^. In the US, these were then incorporated into a nationwide cohort study to differentiate the respective ischemic cardiovascular mortality impacts of PM_2.5_ from different sources, finding that coal combustion particles were approximately five times the health risk for this outcome than other particles^23^. Hence, improving the study design requires not only finer measures of time and spatial exposure resolution, but also to further distinguish pollution constituents/sources. These differentiations are then linked to health outcomes, helping guide the optimal public health strategies and respective regulatory efforts^6^. In the next section, we provide examples where finer resolution monitoring strategies have been adopted, and where air quality policies have been changed as a result.

While not yet numerous, there are examples of approaches that result in highly granular measurement of pollution over large areas. In London, for example, King’s College London “London Air” website provides hourly exposure estimates at a 20 m resolution across London for NO_2_, O_3_, PM_10_ (thoracic PM mass less than 10 µm in diameter), and PM_2.5_^54^, using models relying on data from monitoring networks. With recent advancement and proliferation of low-cost sensor technology, networks of such sensors are also being deployed across urban locations to measure air pollution concentration levels. Examples include deployments of a distributed network of sensors in eight locations in Xi’an, China to measure and locate pollution hotspots^55^ and 32 electrochemical sensor nodes deployed over a 2-month period in Cambridge, UK, collecting high spatial and temporal resolution data on carbon monoxide (CO) concentrations^56^. Researchers also recently carried out sampling at 25 locations in Rochester, NY using low-cost sensors to estimate hourly concentrations of PM_2.5_^57^. As demonstrated by these studies, the low-cost, ease of operation, and the high volume of data generated by sensor networks can allow for streamlined deployment and maintenance of multiple units, providing improved and detailed spatiotemporal coverage in a given area. Cities and government agencies around the world are increasingly engaged in partnerships for the application of sensor technologies^58^, for example, the city of Chicago, in partnership with University of Chicago and Argonne National Laboratory, recently launched Array of Things (AoT), which consists of a network of sensor boxes mounted on light posts to collect a host of real-time data on Chicago’s environmental surroundings (e.g., air quality, temperature, and wind speed) and urban activity. The AoT aims to provide real-time, location-based data to the public, allowing researchers, policymakers, developers, and residents to collaborate and take action.

Other approaches have employed land use regression (LUR) methods to empirically model air pollution levels using both geographic variables and regulatory stationary monitoring data. LUR models that incorporate mobile sampling have also recently gained traction as a cost-effective approach to model urban air quality. Mobile sampling consists of continuous monitors mounted on mobile platforms (e.g., foot, bicycle, and cars) repeatedly sampling travel routes, and then applying statistical approaches to predict air pollution concentration levels. For example, efforts in Hong Kong^59^, Montreal^60^, Minneapolis^61^, and San Francisco^62^ have successfully modeled intra-urban air quality with high-predictive power and increased spatial resolution. For example, a Google Street View mapping vehicle, outfitted with air quality sensors, sampled every street in a 30-km^2^ area of Oakland, CA in order to reveal urban air pollution patterns at 4–5 orders of magnitude greater spatial precision than possible with regulatory central-site ambient monitoring. Vehicle-based mobile measurements were employed to create LUR models to estimate the spatial variation of PM_2.5_ and PM_10_ in the downtown area of Hong Kong. The “OpenSense” project in Zurich, Switzerland^63^ used mobile sensor nodes installed on top of public transport tram vehicles in the city to create pollution maps with a high spatial resolution for ultrafine particles and particle counts. This project aimed to use ultrafine particle maps created with measurements from mobile air pollution monitoring network to build apps to minimize resident’s exposure by providing optimal pollution-avoidance travel routes.

Deployment of improved measurement technologies is now allowing policymakers to target their mitigation and evaluation measures with far greater precision. This happened, for example, in three Israeli cities, which resulted in switching from heavier oil-fueled power plants to cleaner burning natural gas, and a corresponding reduction in mortality^64^. Spatially granular analysis of pollution has been carried out in London, resulting in seminal policy progress in traffic congestion pricing^65^, while bans on diesel fuel have been put in place in many European cities.

**Fig. 2:**
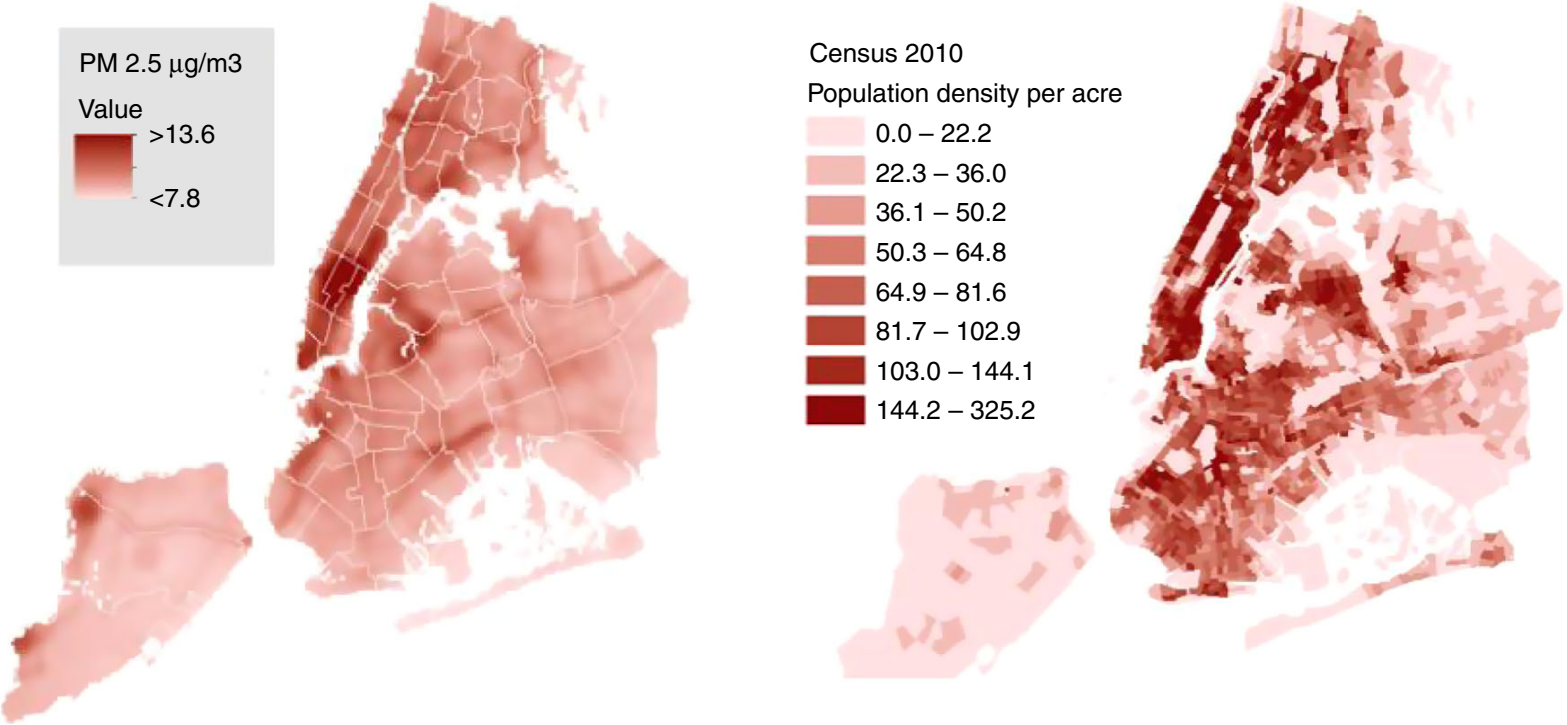
(Left) Yearly average distribution of particulate matter PM_2.5_. Source: New York City Community Air Survey (NYCCAS). (Right) NYC-population density (source American Community Survey, open source online)

To illustrate how improved measurement and policy interact, we cite one particular case in New York City, where a detailed study of patterns of pollution resulted in certain diesel fuels being banned. The 2005–2007 campaign involved the chemical composition analyses of pollution, carried out by the NYC Department of Health and Mental Hygiene (NYC DOHMH)^66^, as part of a program called the New York City Community Air Survey (NYCCAS). In this ongoing study, spatial variations in pollutant concentrations have been measured using 2-week averaged concentrations (Fig. 2). These data were measured using street level monitors that have been methodically rotated among approximately 150 sampling locations around the city^7^, incorporating unusually rich chemical analysis, including PM_2.5_, nitrogen oxides, black carbon, ozone, and sulfur dioxide. Critically, the PM_2.5_ itself was analyzed for constituents, such as aluminum, bromine, calcium, copper, iron, potassium, manganese, sodium, nickel, lead, sulfur, silicon, titanium, vanadium, and zinc. The measured concentrations have been used, along with population density and attributes of the physical infrastructure, as inputs to a LUR model in order to estimate pollutant level and composition at a spatial resolution of 300 m, resulting in the identification of sources (Fig. 2 left). Once the rich details of pollutant concentrations were established at the local level in New York City, it was then possible for researchers to gain insight into the average exposures of those living near particular sources of pollution^67,68^. As a result, in April of 2011, specifically citing the NYCCAS study, New York City Department of Environmental Protection (DEP) set a new rule to phase out the use of two highly polluting forms of heating oil: those known as Number 6 and Number 4.

However, while the broad correlation between adverse respiratory health outcomes and residential proximity to traffic pollution is known in New York City and elsewhere, we are still learning about the individual contributions of different pollutants. In a panel study among inner-city children with asthma^69^, a study was used to evaluate the associations of adverse asthma outcome incidences with increased personal exposure to PM_2.5_ as a whole, and the diesel-related carbonaceous fraction of PM_2.5_ known as EC soot, in particular. The study found increased risk of cough, wheeze, and total symptoms to be associated with personal exposure to increased diesel EC. The findings pinpointed the diesel “soot” fraction of PM_2.5_ as most responsible for pollution-related asthma exacerbations among children living near roadways. This helped identify the particular diesel fuels that were most responsible, for which substitutes were readily available at only marginally higher prices. This led to calls for reductions in these exposures^67,70^, with resulting government actions for less diesel traffic, and limitations on idling vehicles near public schools in NYC. These examples have documented how improved science can lead to more effectively targeted public health action^71^.

Analysis of data from the NYC studies above have shown the within-city variations in air pollution-related premature mortality and morbidity at 42 city neighborhoods, as well as the benefits of the elimination of the low-quality fuel oil^6^. Study results indicated that the complete phase out of the low-quality building fuel oil reduced the annual average level of PM_2.5_ by an estimated 0.71 μg/m^3^ citywide (~0.5% of city-wide mean concentration). This was further estimated to have resulted in an annual decrease of 290 premature deaths, 180 fewer hospital admissions for respiratory and cardiovascular complications, and 550 fewer emergency department visits for asthma. The largest reductions of air pollutant emissions were in more affluent neighborhoods, but the greatest health benefits were estimated to occur in the high-poverty neighborhoods. This disparity in effects is attributed to the higher baseline morbidity and mortality rates at lower income levels. These estimates of the 2005–2007 human toll and cost of PM air pollution exposures are summarized in Table 1. The valuation shown was calculate with BenMAP methodology developed by USEPA, using the events as function of age group from studies carried by NYCDOH^6^. The citywide 3200 premature death can be compared to the 290 decrease in deaths as result of the switch to cleaner fuel.

**Table 1:** Annual health events attributable and cost of PM_2.5_ exposure compared to background levels (2005–2007)

Health effect	Age group	Events	Valuation
Premature mortality	30 and older	3200	$30,772,770,000
Hospital admissions for respiratory	20 and older	1200	$38,000,000
Hospital admissions for cardiovascular	40 and older	920	$34,770,000
Emergency department visits for asthma	Under 18	2400	$1,020,000
Emergency department visits for asthma	18 and older	3600	$1,530,000

## Pollution and policy at the individual level

For all that knowledge and public policy have advanced in recent years, much more needs to be known to further optimize policy priorities and decision-making as related to clean air and public health. In terms of the impact of air pollutant exposure on human health, it is critical to better understand: (1) pollutant exposures at the level of individual and (2) cofactors that have a bearing on health at the level of individual.

It is now recognized that personal exposure to pollution can vary dramatically from block to block in a city, and over time during a day. This cannot be accurately assessed with only the detailed geospatial mappings of fluctuating pollution levels. People move through physical space, and their mobility must be considered. The public spends only a part of their daily lives at home, and there are now technologies available for personal-level exposure assessment to assess the effects of that mobility on their individual exposure profiles^72^. In addition to mobility, even those who live next door to one another may have very different residential pollution exposures, depending on the quality and effectiveness of any in home ventilation or filters. This may depend on the quality of air conditioning, or other unmeasured home-specific factors. Accordingly, the exposures and health implications of pollution may be radically different for population groups living in close proximity to high levels of outdoor pollution. NYC, in particular, is a good illustration of this effect, with many wealthy individuals living near pollution from traffic in Midtown Manhattan, albeit many at high elevation in tall buildings. Yet less is known about exactly why the wealthier population has been found to be less acutely affected by pollution-induced health problems, such as asthma.

It is now technically possible to get most pollution exposures in real time, and to track individual exposures over many years. Acceptance of the benefits of individual-level exposure assessment is growing, and trials including those cited above are underway worldwide, showcasing the value of these advances in technology. In this section, we document ongoing advances in the understanding of personal exposures.

One new insight in relation to pollutant exposure concerns travel to work. It is now known that daily commutes may contribute disproportionately to amount of daily inhalation to air pollutants particularly in urban settings. Designing cities to promote physically active travel is a potential strategy to improve public health, but the built environment can provide both benefits (physical activity) and hazards (air pollution). Exposures to air pollution during active travel were estimated in Minneapolis, Minnesota^73^; active travel occurs on high-traffic streets or near activity centers where concentration levels are highest, and that only 2-3% of blocks have high active travel with low concentrations. The study authors identified 20% of local roads where shifting active travel from high-traffic roads to adjacent low-traffic roads would reduce exposure by around 15%. In another study, exposures and inhaled doses of air pollutants were evaluated in different travel microenvironments in Barcelona, Spain^74^. Travel modes explained much more of commuters’ exposure variability than meteorology, and moving by car experienced highest concentrations for all contaminants, including black carbon, ultrafine particles, carbon monoxide, PM_2.5_, and CO_2_. Whether certain populations may experience different levels of exposure needs to be ascertained through larger scale studies. Whether there are inequalities among different socioeconomic groups for exposure to air pollutants during commuting in London was compared^75^; no systematic relationship between income deprivation and pollutant concentrations was found, suggesting that differences between transport modes are a stronger influence on exposure. People from low-income areas have rely more heavily on the bus for transport, receiving high exposures during commute^75^. A study of 42 healthy adults in Ottawa, Canada found that short-term exposures to traffic pollution may contribute to altered autonomic modulation of the heart in the hours immediately after cycling^76^. Another clear indicator of the importance of mode of transport is illustrated by an example in central London, where the health benefits of walking in highly polluted Oxford Street were negated by exposure to traffic pollution among individuals who are healthy as well as for those with chronic health conditions^77^.

Exercise is also an important factor to consider when evaluating the effects of air pollution exposures, particularly if exercise is done outdoors, as it can increase the dose received at a given ambient concentration exposure. During exercise, increased pulmonary ventilation (i.e., inhaled volume of air per minute) brings more air pollution through the conductive airways than at rest^78^. This can lead to greater dosages of air pollution among exercising individuals, and greater health effects than at lower ventilation rates^79^. Thus, exercise has the potential to increase dose and adverse health effects over nonexercising individuals, and should be considered by the public in choosing where to exercise, as well as in the design and interpretation of air pollution exposure and health assessment studies.

Nevertheless, studies indicate that the benefits of exercise can outweigh the adverse effects of pollution exposure at the population level. Assessment of health impacts of promoting active transportation (increased cycling and increased walking) across six European cities (Barcelona, Basel, Copenhagen, Paris, Prague, and Warsaw) in the TAPAS study found that the potential health benefits outweighed the risks posed by increased inhalation of air pollution^80^. The trade-off between exercise benefits and air pollution harms, however, is dependent on city characteristics (e.g., built environment and pollution levels), as well as the individual and their activities (e.g., health status, mode of transport, and locale of exercise).

Improved characterizations as to how people move through time and space will improve the understanding of heterogeneities in population exposure. A recent study^81^ used cell-phone data to estimate the mobility of several million people in New York City and then used this, along with data on spatiotemporal PM_2.5_ concentration levels, to calculate the population-weighted pollution exposure, and compared to “Home Population Exposure”, which assumed a static population distribution. The value of this dynamic approach was demonstrated in that the temporal variability of the cell phone -based exposures was significantly different to “static” estimates based on Census data, during both the daytime and the nighttime.

In another study^82^, survey-based data were used to derive a model of travel behavior and time-activity patterns for 89,385 subjects in Hong Kong. This information on population mobility was then incorporated in LUR models to estimate dynamic time-weighted air pollution exposure for different age, sex, and employment groups. Working adults and students showed increased exposure, due to their higher mobility, suggesting that population mobility patterns add value to epidemiological studies of air quality and human health.

Advances have also been made in the development of citizen-based monitoring technologies to assess location-specific or personal-specific exposures. “Participatory sensing” refers to a methodology in which sensors are carried by citizen volunteers, who often benefit in terms of access to data on their own exposures. For example, one study explored the potential of “opportunistic” monitoring to map the exposure to air pollution in the urban environment at a high-spatial resolution^83^. The sampling design made use of an existing mobile infrastructure or people’s daily routines to move measurement devices around. The study equipped the city wardens in Antwerp, Belgium, who are outdoors for a large part of the day on surveillance tours by bicycle or on foot, with black carbon monitors. The data collection gathered a total of 393 h of measurements to capture and characterize the pollution within those patrolled areas of the city. CITI-SENSE is another initiative launched in Europe to develop “citizens’ observatories” to “empower citizens to contribute to and participate in environmental governance, to enable them to support and influence community and societal priorities and associated decision-making.” CITI-SENSE enables people to use low-cost air quality monitors in eight European cities to collect and share their data^84^. One of the largest community-based air monitoring networks in the US is the Imperial County Community Air Monitoring Network, with 40 sensors generating real-time PM sensors^85^. Similar community-driven, citizen-science efforts by a collaborative group of community, academic, nonprofit, and government partners could supplement regulatory data, as well as drive public policy and discourse. Notably, the State of California recently passed new legislation (SCAQMD AB 617) requiring air districts to establish “community air monitoring systems” that include community input and low-cost sensors, the first regulation of its kind in the world^86^. Globally, many nonprofit organizations and agencies are beginning to share their air pollution concentration data as part of “open data” and “citizen science” movements. Other examples include determination of burden of disease at a given location using estimation of pollution at high spatial and temporal resolution (see animation showing one day in NYC), along with passive sensing of human mobility using cellular phone signals^87^. Cities and agencies worldwide are beginning to share their environmental and exposure data: the nonprofit AirCasting is example of a crowd-sourced air pollution mapping platform using its mobile monitor “AirBeam,” with more than 2 million data points shared so far. Similarly, PurpleAir shares online both personal and central-site air quality data from around the world.

## Studies on real-time personal exposure and health

The individual-level health outcomes due to short- and long-term exposure in relation to baseline health, SES, and other key cofactors is emerging as the focus for future research. As indicated above, it is now possible to greatly increase the granularity of data on geospatial patterns of pollution, and combine this with detailed information on individual exposures. In principle, it should soon be possible to more accurately gauge the beneficial effects of well-filtered air in the home and office, living on a tree lined street, limiting outdoor exposure for children on polluted days, or avoiding exposure to rush hour traffic conditions. It is this more individuated knowledge that would advance the understanding of the biological mechanisms of response, and give rise to changes in individual and social behavior to reduce air pollution exposures, and motivate protective public policies.

The potentially massive public health benefits that granular data would provide is motivating research efforts worldwide. For example, an increasing number of studies have used temporally resolved exposure data to estimate longer term exposures (e.g., weekly exposures throughout pregnancy). The EU funded EXPOsOMICS project^88^ aims at generating this type of space and time resolved exposure data.

The goal of much of this recent research on pollution exposure and health outcomes is to move beyond group associations, and investigate causal pathways and effects at the individual level. The questions asked include: “Who is most affected by air pollution, and why?”. This is challenging to answer in standard data sets, in which exposures and/or characteristics are grouped. This is why the recent studies outlined above (for example, the work^47^ on inversions in Sweden) are of such importance. Yet, valuable as these exposure-effects pathway studies are, they cover relatively short periods of time, using aggregate population groups, and only highly specific effects. There are no comprehensive studies that track exposures over time and link measured exposures to the wide range of related health outcomes on the individual level, both short-term and long-term^89^. For example, participant exposures for entire residence and work location histories after enrollment to prospective cohorts are lacking in most available studies today, which could be addressed in future studies. It should be acknowledged that short-term peaks in exposure are most pertinent when targeting illness (e.g., asthma) exacerbations, requiring temporally and spatially resolved exposure data; while long-term annual or decadal averages are pertinent to the development of diseases (e.g., cardiovascular disease (CVD)). Thus, study platforms that enable the integration of these two scales, at level of the individual, promise to advance the understanding of the respective roles of short-term exposures on acute illness, while delivering realistic measures of cumulative exposures’ role in chronic disease development.

In addition to measuring pollution-related health impacts, such as respiratory and CVDs, it is also important to measure baseline health conditions known to impact susceptibility to pollution-related health outcomes. These conditions themselves have been found to be SES dependent, suggesting the need to study interactions among exposure, health, and socioeconomic status over longer periods. In addition to socioeconomic factors, there is some evidence of psychological effects of pollution, for example on mood states, which are rarely monitored^90,91^. For this reason, research groups worldwide are pushing for enriched measurement of pollution exposures and their many interactions with the human condition.

There are exposure assessment approaches that could help address such pollution-health interaction issues. As noted above, the portability and mobility of sensor technologies allow for measurements of real-time exposures to airborne pollutants. Similarly, deployment of wearable technologies and equipment assessing real-time health outcomes is a growing area of research. Researchers have evaluated the effects of acute PM_2.5_ exposure during different modes of transport (cycling, walking, bus, and train) on changes in heart rate variability (HRV) in 32 young participants. HRV was measured using Actiheart units placed on participants’ chests. Personal PM exposure was monitored using mobile real-time monitors carried by participants (Met One Aerocet 531 particle profilers were used to measure PM at 2-min intervals). Elevated PM_2.5_ and PM_10_ inhaled and lung deposited doses were significant associated with decreased HRV indices. Percent declines in standard deviation of normal RR heart beat intervals were stronger in cyclists and pedestrians, in comparison to bus and train passengers^92^. Another study assessed association between PM_2.5_ exposure and HRV in 18 healthy male Swiss highway workers^93^. The workers were equipped with small devices to record ECG (H12 + Digital) and personal-level PM_2.5_ (DataRAM PDR100, a light scattering nephelometer). Acute exposure to PM_2.5_ was followed by reduced HRV, and reduced modulation of HRV, were also observed at elevated PM_2.5_ levels for all three activity phases assessed (work, home, and night).

Other parallel efforts to monitor personal pollution exposures include a multicity study in Europe^94^ that measured movement, black carbon air pollution, and physiological health markers of 122 adults for 3 weeks in 3 European cities (Antwerp, Barcelona, London) as part of the Physical Activity through Sustainable Transport Approaches project. A SenseWear for physical activity registration and a Zephyr BioHarness 3 was used to measure participants’ real-time breathing rate, breathing wave amplitude, and heart rate. MicroAeth black carbon monitors (AE51) were used to measure personal exposures to traffic related air pollutants. The study compared minute ventilation and inhaled dose across methods in a panel study design. There are also multiple efforts ongoing in the U.S.: the NIH launched the Pediatric Research using Integrated Sensor Monitoring Systems (PRISMS) program, a multi-institution center, in 2015 to develop sensor-based, integrated health monitoring systems for measuring environmental, physiological, and behavioral factors in pediatric epidemiological studies of asthma. Another ongoing effort by city of Louisville, Robert Wood Johnson Foundation, and Propeller Health, a company that collects data from sensors attached on asthma inhalers, looks to identify environmental triggers associated with inhaler use. In that study, several environmental factors were significantly associated with inhaler use, including the Air Quality Index, PM_10_, weed pollen, and mold^95^.

## The New York City Human Project

There is also now a prototype project beginning in New York City that will measure individual exposures over extended period of time (years), with plans to link these exposures to health and individual behavior. “The Human Project” (THP), presently in its pilot stage, is designed to provide a longitudinal study of the relation between pollution and health, and socioeconomic factors, behavior patterns, and other cofactors^96^. The goal of THP is serve as a resource for the discrimination of individual-level variability in the effects of air pollution on health, and associated socioeconomic modifiers. To ensure sufficiently rich measurement of human exposure, THP is being designed as a long run panel study. In its full-scale implementation, it is expected to gather data on a representative sample of 10,000 New Yorkers in their family units with plans to track this panel over 20+ years. Already in place are key elements of the framework for participation, privacy, security, collaboration, resource sharing, and dissemination. Central to the research design is the collaboration of researchers from multiple disciplines, including environmental medicine, economics, psychology, neuro-economics, urban and environmental engineering, data analysis and modeling, working to put together a common analytic framework. The aim in this work is the rapid development and deployment of new methods of pollution exposure assessment, while increasing the duration of analysis (therefore the number of sampling points per individual).

Individual-level health data will be central to THP and its study of pollution. This will include the participants’ medical data such as electronic medical records (EMRs) data, health insurance claims, in-patient and outpatient hospital visit data. Data sharing agreements are currently underway with EMRs data providers and the New York State Department of Health (NYSDOH), and strict protocols for data privacy and security are built into the study. Other EMRs will be acquired from the New York State (NYS) Health Information Exchanges (HIEs). Overall, this project seeks to make corresponding advances in the understanding of the links between exposure, health, wealth, and human behavior, focusing on the New York City area as the laboratory.

Information on THP participants’ hospital inpatient stays and outpatient visits will be captured from NYS’s Statewide Planning and Research Cooperative System (SPARCS). SPARCS is a comprehensive all payer data reporting system established in 1979 as a result of cooperation between the health care industry and government. SPARCS data includes individual-level detail on patient characteristics, diagnoses and treatments, services, and charges for each hospital inpatient stay and outpatient visit. This includes ICD 9/10 codes and data on ambulatory surgery, emergency department, and outpatient services.

Data on participant medical encounters covered by Medicaid will be also obtained from the Medicaid Data Warehouse (MDW). This contains over 25 years of structured claims data and allows authorized users to extract longitudinal information on an individual’s encounters with the Medicaid system. This data is also made available for authorized purposes, through agreements with NYSDOH.

In addition to having detailed information from medical records, advances made by the research community quantifying individual exposure (described in previous section) will be used to link exposure to short- and long-term health outcomes. Mobile sensors and applications will be used to record a host of physiological metrics of participants’, such as activity levels, pulse, and heart rate variability, in real time. A mobile smartphone “app” will be used to push surveys to participants’ mobile devices to measure fluctuations in mood, perception of air quality and symptoms such as shortness of breath.

THP will measure behaviors in a granular manner. This will allow the study to gauge the prevalence and effects of so-called avoidance behaviors in lowering the adverse effects of air pollution on health^97^. Such activities include purchasing preventive pharmaceuticals and reducing time spent in polluted environments^98^. To date, little is known about how prevalent and important these behaviors are. For that reason, THP is expected to record a rich battery of behavioral responses to pollution.

This projects and similar efforts carried out in Europe are beginning to successfully interject personal environmental and health monitoring into the study and evaluation of the human health effects of air pollution. However, challenges remain. While sensors enable us to monitor pollution at a highly granular scale, this benefit is somewhat offset by the less accurate/reproducible measurement these devices offer, as smaller and/or cheaper devices tend to be less sensitive, less precise, and less chemically specific to the compound or variable of interest. Thus, while ambulatory devices give a very detailed picture of individual exposure, we still struggle to implement these approaches at a scale required to have sufficient power to assess the more subtle, but widespread, impacts of air pollution exposure.

## Conclusions

This paper outlines the current state of research on spatial mapping of air pollution exposures, and the quantitative analysis of their human health effects. It highlights the interaction of science with policy and the many places advances in scientific understanding have produced appropriate policy responses. We outline newly feasible approaches to measuring exposures and health outcomes over the course of the human life cycle in their economic, geographic, familial, and social context. As such extensive new data becomes available, policymakers will be tooled to pinpoint and eliminate key environmental hazards. Such policies are particularly necessary to protect those who are most vulnerable, including the young, the old, and the less well-off.

We have documented examples of ways in which scientific progress has resulted in public policy improvements, and the opportunities and technical feasibility of increased assessment of individual exposures and health effects. We end with a description of a prototype study designed for the assessment of air pollution exposures and effects over an individual’s life cycle. It is possible that such air pollution research will have policy outcomes as strong as those that followed research on the health impacts of smoking. In that case, scientific awareness of its damaging effects gave rise to public health initiatives that resulted in dramatic declines in smoking-related deaths in the developed world^99,100^. Along these lines, the recent First WHO Global Conference on Air Pollution and Health in Geneva, Switzerland (30 October–1 November 2018) highlighted the need to make commitments to air quality around the globe. By personalizing the exposures and impacts, such studies can contribute to this advancement of environmental health science and policy, and thereby have the potential to engender more optimal societal changes to the benefit of everyone, perhaps particularly for society’s most adversely affected individuals and groups.

## Supplementary information

Description of Additional Supplementary Files

Supplementary Movie 1

## Data Availability

Figure 1, created using open source data: OpenNYC: https://data.cityofnewyork.us/City-Government/Community-Districts/yfnk-k7r4 Data2go.nyc: https://data2go.nyc/faq.pdf Figure 2, created using open sourced data: Figure 2 (left): https://data.cityofnewyork.us/Environment/NYCCAS-Air-Pollution-Rasters/q68s-8qxv Figure 2 (right): https://www1.nyc.gov/site/planning/data-maps/nyc-population/census-2010.page, and using Census Tract shapefile, also available open source: https://www1.nyc.gov/site/planning/data-maps/open-data/districts-download-metadata.page Data and code for the animation of hourly 300 m spatially resolved concentration of PM2.5 is available at: https://github.com/masoudhub/Nowcasting.git
