# Supplementary material for: Advancing environmental exposure assessment science to benefit society
Source: Nat Commun. 2019 Mar 15;10:1236. doi: 10.1038/s41467-019-09155-4 (PMC6420629; doi:10.1038/s41467-019-09155-4)
Supplement: Supplementary file 1 — Description of Additional Supplementary Files [file 41467_2019_9155_MOESM1_ESM.pdf]

Description of Additional Supplementary Files

File Name: Supplementary Movie 1

Description: Hourly estimation of particulate matter (PM2.5) concentration in NYC using kriging technique incorporating seasonal spatial (300m) distribution of the particulate matter, and hourly variation of the concentration from 11 regulatory stations in NYC. Acknowledging work by Milad Aghamohamadnia and Te Du. Source data and algorithm available on Github: https://github.com/masoudhub/Nowcasting.git
